# Insights Into the Coinfections of Human Immunodeficiency Virus-Hepatitis B Virus, Human Immunodeficiency Virus-Hepatitis C Virus, and Hepatitis B Virus-Hepatitis C Virus: Prevalence, Risk Factors, Pathogenesis, Diagnosis, and Treatment

**DOI:** 10.3389/fmicb.2021.780887

**Published:** 2022-02-03

**Authors:** Sagarika Shahriar, Yusha Araf, Rasel Ahmad, Pravakar Kattel, Ganga Sagar Sah, Tanjim Ishraq Rahaman, Rahila Zannat Sadiea, Shahnaj Sultana, Md. Sayeedul Islam, Chunfu Zheng, Md. Golzar Hossain

**Affiliations:** ^1^Biotechnology Program, Department of Mathematics and Natural Sciences, BRAC University, Dhaka, Bangladesh; ^2^Department of Genetic Engineering and Biotechnology, School of Life Sciences, Shahjalal University of Science and Technology, Sylhet, Bangladesh; ^3^Department of Microbiology and Hygiene, Bangladesh Agricultural University, Mymensingh, Bangladesh; ^4^Department of Biotechnology and Genetic Engineering, Faculty of Life Sciences, Bangabandhu Sheikh Mujibur Rahman Science and Technology University, Gopalganj, Bangladesh; ^5^Department of Biological Sciences, Graduate School of Science, Osaka University, Osaka, Japan; ^6^Department of Microbiology, Immunology and Infectious Diseases, University of Calgary, Calgary, AB, Canada

**Keywords:** coinfection, HIV, HBV, HCV, prevalence, risk factors, pathogenesis

## Abstract

Human immunodeficiency virus, hepatitis B virus, and hepatitis C virus are three blood-borne viruses that can cause major global health issues by increasing severe morbidity. There is a high risk of coinfection with these viruses in individuals because of their same transmission routes through blood using shared needles, syringes, other injection equipment, sexual transmission, or even vertical transmission. Coinfection can cause various liver-related illnesses, non-hepatic organ dysfunction, followed by death compared to any of these single infections. The treatment of coinfected patients is complicated due to the side effects of antiviral medication, resulting in drug resistance, hepatotoxicity, and a lack of required responses. On the other hand, coinfected individuals must be treated with multiple drugs simultaneously, such as for HIV either along with HBV or HCV and HBV and HCV. Therefore, diagnosing, treating, and controlling dual infections with HIV, HBV, or HCV is complicated and needs further investigation. This review focuses on the current prevalence, risk factors, and pathogenesis of dual infections with HIV, HBV, and HCV. We also briefly overviewed the diagnosis and treatment of coinfections of these three blood-borne viruses.

## Introduction

The coinfection of viruses can be a serious public health problem because most medicines are designed to control and manage a single infection. Acquired immunodeficiency syndrome (AIDS) caused by the human immunodeficiency virus (HIV) is one of the most important and prevalent disease conditions that has been spread among humans for the last two decades (Kim et al., 2000). More than 75 million people worldwide have been infected with HIV, and approximately 37 million individuals are currently living with this infection. Hepatitis B (HB) and hepatitis C (HC) viral infections are highly prevalent among HIV-infected individuals due to sharing the same transmission routes. Hepatitis caused by both hepatitis B virus (HBV) and hepatitis C virus (HCV) leads to severe liver disorder, and morbidity and mortality are now increasing due to coinfection with HIV (Easterbrook et al., 2012). Coinfection with HIV can modify the natural characteristics of HBV by genome replication status, higher rates of chronic infection, and liver disease progression (Ndifontiayong et al., 2021). The impact of HIV on HBV is critical, as HIV can provoke chronic HBV infection, which can lead to hepatocellular carcinoma (HCC) (Maponga et al., 2020). While in HIV-HCV coinfection, HIV increases the HCV viral load and accelerates liver disease progression (Rodrigo, 2020). HIV, HBV, and HCV are transmitted *via* blood, shared needles, syringes, and other injection equipment, sexually, or even from pregnant mothers to babies (Pfaender et al., 2016). The hepatotropic viruses, both HBV and HCV, attack the liver cell and cause inflammation. However, HIV can attack any targeted cell in the mucosal tissue and spread through the whole lymphoid system (Siebers and Finlay, 1996). As a result of shared transmission routes, HBV, HCV, and HIV can easily cause coinfection, more pervasive than an infection caused by either HBV or HCV.

On the other hand, patients dually infected with HCV and HBV carry a significantly higher risk of developing fulminant hepatic failure, liver cirrhosis, and HCC than those with HCV or HBV infection alone (Liu, 2014). A recent study showed that viral infections, such as HBV, HCV, and HIV, are the second most common cause of morbidity and mortality among patients with thalassemia (Bhuyan et al., 2021). Great attention should be paid to the stages of liver disease, virus predominance, and the presence of HIV infection and comorbidities to conduct a better treatment. Several other therapies are being assessed in these circumstances, along with highly active antiretroviral therapy (HAART) (Zhang et al., 2014). However, proper treatment for patients suffering from dual coinfection, either with HIV, HBV, or HCV, is yet to be discovered. The current review is focused on various aspects of HIV, HBV, or HCV coinfection, with a special emphasis on prevalence, risk factors, pathogenesis, diagnostic markers, and treatment.

## Structure and Replication of Human Immunodeficiency Virus, Hepatitis B Virus, and Hepatitis C Virus

### Human Immunodeficiency Virus

**FIGURE 1:**
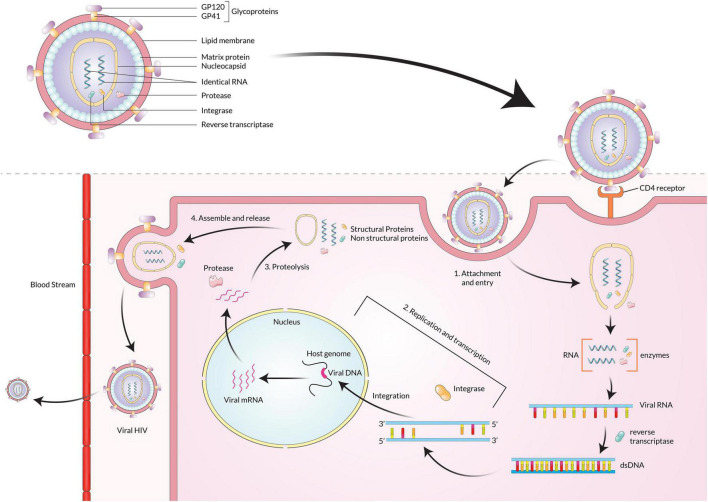
After HIV GP120 binds with the receptors on the CD4 cell surface, the viral particle fuses with the membrane and enters the cell. The HIV genome ssRNA is reverse transcribed into the dsDNA using reverse transcriptase. The viral DNA is then integrated with the host cell DNA using integrase. The various structural and non-structural proteins are produced from the integrated DNA, and then viral particles are assembled and released from the cell.

Human immunodeficiency virus infects humans with two types of lentiviruses of the Retroviridae family. The structure of HIV is roughly spherical with a diameter of about 120 nm and covered by an outer lipid envelope membrane (de Jongh et al., 1992). The viral genome consists of two identical single-stranded RNA molecules enclosed within the core of the virus particle along with three viral enzymes-reverse transcriptases (RT/RNAse), integrase, and protease (Bodsworth et al., 1991). The initial steps of infection are dependent on protein-protein interactions, where the surface protein GP120 of the virus binds to the CD4 receptors on host cells (Xiao et al., 2008). The attachment causes a conformational change by which channels are formed. Due to its high hydrophobicity, the virus particle is engulfed by the target cell’s plasma membrane, and the reverse transcriptase enzyme transcribes the single-strand RNA genome into double-stranded DNA (dsDNA) (Ockenga et al., 1997). The dsDNA is then transported through the nucleopore complex into the nucleus and integrated within the host genome by the integrase enzyme, producing mRNA and translating it into structural and non-structural proteins (Figure 1). These proteins are then assembled to form viral progeny and finally released into the bloodstream (Yim and Lok, 2006; Miailhes et al., 2007).

### Hepatitis B Virus

**FIGURE 2:**
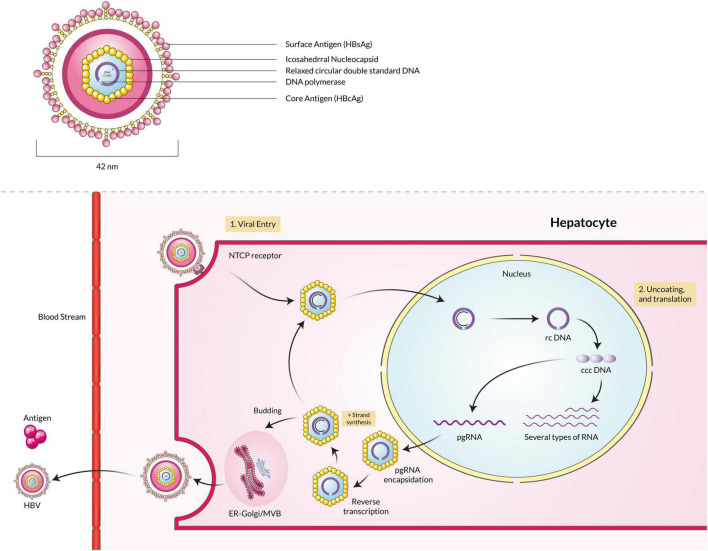
Hepatitis B virus particle binds with the NTCP receptor, fuses with the membrane, and enters the host cell. The rcDNA is converted into cccDNA, transcribed into pgRNA, and finally packaged into the capsid. The capsid is enveloped by the ER-Golgi/MVB and released into the extracellular space.

Hepatitis B virus is a member of the Hepadnavirus family, infecting humans, whereas orthohepadnaviruses and avihepadnaviruses infect mammals and birds, respectively (Schaefer, 2007). The infectious virion is approximately 42 nm in diameter with an internal icosahedral symmetry core particle nucleocapsid. The viral genome is a double-stranded DNA around 3.2 kb in size (Ott et al., 2012). The positive strand is the incomplete inner one, and the negative strand is the completed outer one, and viral polymerase is linked to the 5′ end of the minus strand (Bosch et al., 1988; Hossain et al., 2020). The envelope is also the primary structure embedded with the surface antigens (HBsAg), which are the core components of detecting the presence of HBV infections by commercial immunoassays (Hossain and Ueda, 2017). In the first phase of infection, the virion attaches to host cells *via* sodium taurocholate co-transporting polypeptide (NTCP) that helps itself get uncoated by fusion (Yan et al., 2012; Li, 2015). Secondly, upon infection, the rcDNA of the virus is converted into plasmid-like covalently-closed circular DNA (cccDNA) inside the host cell nucleus (Figure 2). Finally, the cccDNA templates several genomic and subgenomic RNAs transcribed by cellular RNA polymerase II. The pregenomic RNA (pgRNA) is then selectively packaged into progeny capsids, and most of the pgRNA reversely transcribed into DNA within the capsids. Then the progeny viruses are released from the cells after being enveloped in the ER-Golgi/MVB (Lee et al., 2008; Hossain et al., 2020).

### Hepatitis C Virus

**FIGURE 3:**
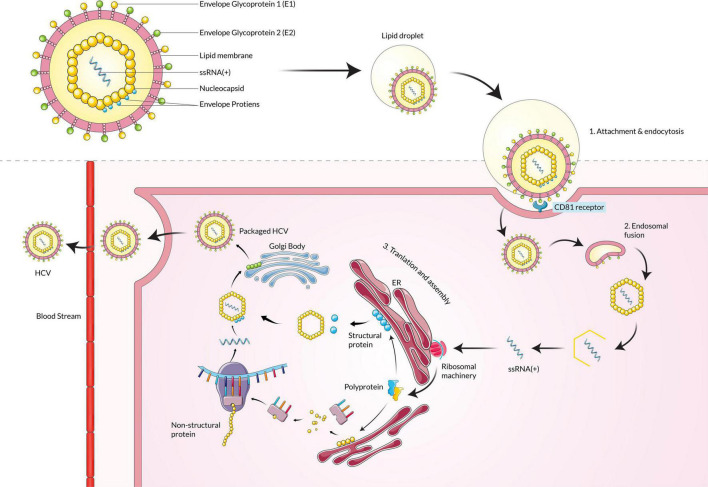
Hepatitis C virus particle binds with the CD81 receptor, fuses with the membrane, and enters the host cell. Upon entering, the ssRNA(+) is translated by ribosomal machinery and produces both structural and non-structural proteins, which assemble and mature in the Golgi body and are then released into extracellular space.

Hepatitis C virus is an enveloped virus that is a member of the genus Hepacivirus under the family Flaviviridae. The genome of the HCV is a positive-sense single-stranded RNA (ssRNA), around 9.6 kilobases in size (Kato, 2000). The ssRNA(+) is protected by a protein nucleocapsid and a lipid envelope membrane (Bukh et al., 1994). HCV infection into the host cells occurs by receptor-mediated endocytosis (Blanchard et al., 2006; Karangelis et al., 2010). The HCV particle binds with the hepatocyte surface molecules such as glycosaminoglycans, CD81, scavenger receptor class B type I (SR-BI), members of the claudin family (CLDN1, 6 and 9), and mannose-binding lectins DC-SIGN and L-SIGN (Barth et al., 2006; Helle and Dubuisson, 2008). Lipoproteins metabolism is also involved in the HCV attachment and replication steps (Grassi et al., 2016). The nucleocapsid is released in the cell cytoplasm from the virus-host cell molecule complex, and then the nucleocapsid is decapsidated to free the ssRNA(+) into the host cytoplasm. Due to its positive-sense properties, the viral RNA acts as mRNA and is directly translated by cap-dependent ribosomal machinery (El-Hage and Luo, 2003). A single polyprotein is translated from the mRNA, later processed to produce several structural and non-structural viral proteins. The six non-structural proteins are NS2, NS3, NS4A, NS4B, NS5A, and NS5B, which help in functions related to viral replication and are involved in host cell pathogenesis (Bartenschlager et al., 1994; Reed et al., 1995; Egger et al., 2002). The non-structural proteins help to make ssRNA(+) by using a ssRNA(–) template that is replicated by NS5B RNA-dependent RNA polymerase beforehand from one ssRNA(+) (Gosert et al., 2003). Finally, the assembled viral particle is incorporated with lipid droplets and released by one of the two predicted pathways such as very-low-density lipoproteins (VLDL) secretory pathway or endosomal sorting complex required for transport (ESCRT) pathway (Figure 3; Penin et al., 2004; Syed et al., 2010; Dubuisson and Cosset, 2014).

## Prevalence of Coinfection

### Human Immunodeficiency Virus-Hepatitis B Virus

It is estimated that approximately 240 million people have chronic HBV infection (Sun et al., 2014). According to WHO, 37.5 million people were estimated to be living with HIV at the end of 2020. Because of shared routes of transmission, HBV coinfection among HIV-positive persons is common. In some regions, over two-thirds of HIV-infected persons have been reported with a history of HBV infection, which means 2–4 million people have HBV-HIV coinfection (Alter, 2006). However, the prevalence varies from region to region among HIV patients with chronic HBV infection (5–10%) due to local endemicity and acquisition mode. In western countries, the prevalence of HIV-HBV coinfection is estimated to be approximately 20% due to being acquired in adulthood, either by drug injection or through sexual transmission (Nyirenda et al., 2008). However, in Asia and sub-Saharan Africa, the endemicity is intermediate to high with 10–20% prevalence, acquired primarily in the perinatal period and early childhood (Lee et al., 2008; Nyirenda et al., 2008). In the United States, it is estimated that half of all patients with HIV infection have been exposed to HBV, which is 20 times higher than in the general US population. In the early 2000s, around 8% tested positive for hepatitis B surface antigen (HBsAg) or had detectable HBV DNA levels (Spradling et al., 2010). In a study conducted in Iran, the HIV-HBV prevalence in the general population and health care workers was very low. The highest prevalence was observed among injecting drug users at 1.88% and prisoners (0.13%) (Table 1; Bagheri Amiri et al., 2016).

**TABLE 1:** Regional prevalence of HIV-HBV, HIV-HCV, and HBV-HCV coinfection.

Coinfection	Region	Prevalence of risk factors among the cases	References
HIV-HBV	Globally	2–4 million people have HBV-HIV coinfection	Alter, 2006
	Western countries	20% coinfection occurs due to drug injection and sexual intercourse	Nyirenda et al., 2008
	Asia and Africa	10–20% coinfection occurs during the perinatal period and early childhood	Lee et al., 2008; Nyirenda et al., 2008
	United States	Half of the HIV infected patients are coinfected with HBV	Spradling et al., 2010
	Iran	The prevalence of coinfection is higher in drug users (1.88%) and prisoners (0.13%) than general people	Bagheri Amiri et al., 2016
HIV-HCV	Globally	20–30% of 3.5 million HIV patients are infected with HCV	Hernandez and Sherman, 2011
		The prevalence of HIV-HCV coinfection among intravenous drug users is estimated to be around 90%	Sherman et al., 2002
HBV-HCV	Globally	10–15% of patients with chronic HBV infection are infected with HCV	Crespo et al., 1994; Ohkawa et al., 1994; Senturk et al., 2008
		2–10% of anti-HCV-positive patients are HBsAg positive	
	India	The prevalence of HBV-HCV dual infection is 16%	Chakravarti et al., 2005
	Egypt	The prevalence of coinfection is 0.7%	Mekky et al., 2013
	United States	The prevalence of coinfection is 1.4%	Semnani et al., 2007

### Human Immunodeficiency Virus-Hepatitis C Virus

Globally 37.5 million people live with HIV infection, and approximately 20–30% are infected with HCV (Hernandez and Sherman, 2011). As mentioned above, in the same transmission route, the prevalence of HIV-HCV coinfection among intravenous drug users is estimated to be around 90% (Sherman et al., 2002). According to various reports, approximately 21% of adults with HIV tested positive with HCV infection (Yee et al., 2000). Besides percutaneous drug exposure, coinfection can also be seen in hemophiliacs who received contaminated blood without routine serological screening (Di Martino et al., 2001). HCV is estimated to have a 10-fold higher risk of transmission than HIV through percutaneous routes; hence coinfected individuals are usually first infected by HCV (Kingsley et al., 1987). HCV is spread less efficiently by sexual transmission than HIV (Thomas et al., 1995; Gupta, 2013). The number of HIV-infected people chronically infected with HCV worldwide is approximately 2.3 million (Table 1; Garg et al., 2014).

### Hepatitis B Virus-Hepatitis C Virus

Due to the lack of large-scale population-based studies, the exact number of HBV-HCV coinfected patients is unknown; however, some regional studies have been done. One study reported that the prevalence of HBV-HCV dual infection is 16% in India (Chakravarti et al., 2005), while another reported the prevalence of coinfection is 0.7% in Egypt (Mekky et al., 2013). Data from different regions concluded that approximately 10–15% of patients with chronic HBV infection are also infected with HCV; on the other hand, about 2–10% of anti-HCV-positive patients are HBsAg positive (Crespo et al., 1994; Ohkawa et al., 1994; Senturk et al., 2008). Tyson et al. estimated that the prevalence of HBV-HCV coinfection was 1.4% in the US (Table 1; Semnani et al., 2007).

## Risk Factors of Coinfection

### Human Immunodeficiency Virus-Hepatitis B Virus

**FIGURE 4:**
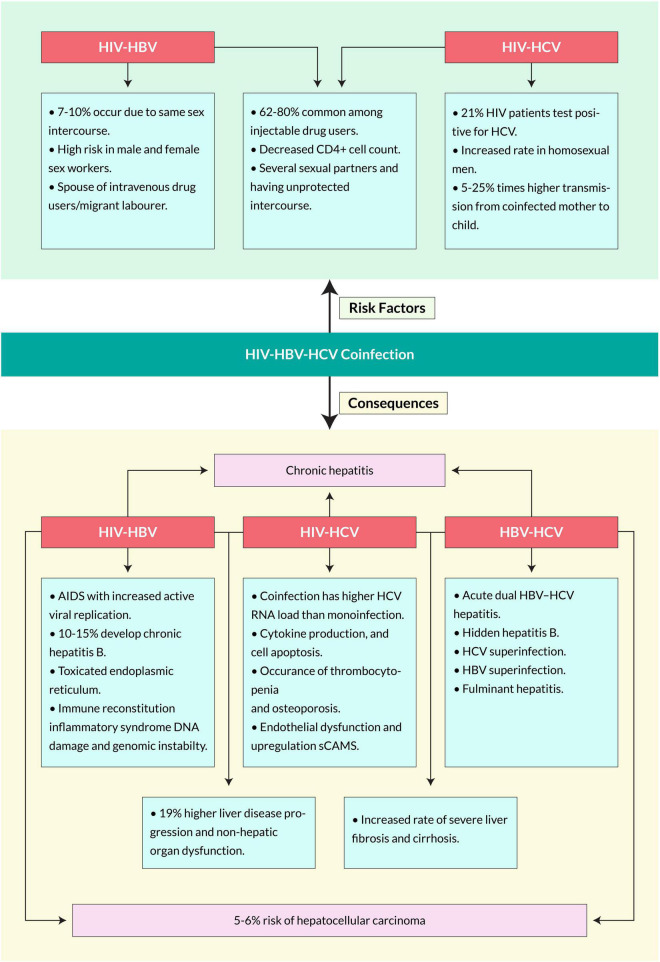
Major risk factors and consequences of HIV, HBV, and HCV coinfection.

The risk factors for coinfection with HBV in HIV-positive individuals may vary in several aspects, including patient age, body mass index, sex, geographical location, current ART regime and duration, key population category, HIV viral load, marital status, and CD4+ T cell count. An epidemiological profile study of HBV-HIV coinfected people conducted in Nepal portrayed some risk factors, including being a spouse of a migrant laborer, a male or female sex worker, an intravenous drug user, or having an HCV-positive status (Bhattarai et al., 2018). Several studies reported that the prevalence of HIV-HBV coinfection among people who inject drugs (PWID) increases with age (Falade-Nwulia and Thio, 2011; Wing, 2017). Due to the chronic and sometimes asymptomatic nature of HIV and HBV infections, the individuals are likely at an even higher risk of chronic progression given the combined effects of age and related immune system dysfunction (Abdala et al., 2008). It is hypothesized that HIV-positive individuals are more likely to be infected with the hepatitis B virus (HBV) than HIV-negative individuals, possibly due to common risk factors. In addition, immunosuppression induced by HIV infection may cause reactivation or reinfection in those previously exposed to HBV (Thio et al., 2002). Other reports showed that coinfection prevalence was higher among men than women, especially in homosexual men, non-Hispanics than Hispanics, and among patients aged 35–44 than younger or older (Porras-Ramírez and Rico-Mendoza, 2020; Figure 4).

### Human Immunodeficiency Virus-Hepatitis C Virus

Human immunodeficiency virus-driven viral hepatitis exacerbates hepatic lesions and increases the transmission of HCV. Several risk factors associated with the HIV-HCV coinfection are shown in Figure 4. Some populations among HIV-positive patients worldwide have more frequent exposure to HCV infection due to engagement in high-risk behaviors, weak family and social support systems, and inadequate access to healthcare services (Health Policy Project, 2014). Coinfection risk factors also vary on epidemiological subjects such as age, marital status, sex, geographical location, HIV viral load, and CD4+ T cell count. In a Nepalese study, HIV-HCV coinfection was higher in individuals >40 years of age. It is more evident in homosexual men with multiple sexual contacts without a preservative (Götz et al., 2005). In a cohort, the incidence of HCV infection among HIV seropositive MSMs increased 10-fold (van de Laar et al., 2007). Moreover, HIV-positives with CD4+ T cell counts of >200 cells/μL were associated with a lower risk of coinfection with HCV. The study suggested that age and CD4+ T cell count may affect the risk for HIV-HCV coinfection (Bhattarai et al., 2018). Furthermore, the blood-borne transmission of the coinfection is 62–80% common among injection-drug users (Yehia et al., 2014). Several studies suggest that HIV and hepatitis transmission are associated with high-risk injection practices such as injection with a syringe previously used by another PWID (Ray Saraswati et al., 2015; Buchanan et al., 2017; Kilonzo et al., 2018). Individuals living with HIV-HCV coinfection are less likely to clear acute HCV infection and more likely to transmit the virus, particularly PWID who share used needles (Shapatava et al., 2006). In the case of mother-to-child, the risk of transmission to the fetus in coinfected women for HIV is between 5 and 25% times higher than in mothers infected by only HCV (Zanetti et al., 1999). Coinfected patients have higher HCV RNA loads and experience more rapid progression of HCV-related liver disease than those without HIV infection (Thomas et al., 2000). HIV infection was found in 13% of hemophiliacs coinfected with HIV and HCV, while HCV infection was detected in just 3% of 162 female sexual partners (Eyster et al., 1991). Other transmission routes, such as sexual intercourse or vertical transmission, have less risk of transmission than percutaneous exposures. The reported prevalence of sexual transmission of HCV-positive individuals is 2–8% and in newborn babies is 2–5%, but three times higher if the mother is HIV positive (Operskalski and Kovacs, 2011).

## Pathogenesis of Coinfection

### Human Immunodeficiency Virus-Hepatitis B Virus

The impact of HIV infection on HBV-induced hepatitis progression is significantly different from HBV infection on HIV disease progression. Around 10–15% developed chronic hepatitis B, which is less likely to be eliminated. HIV-HBV coinfection causes several symptoms, some of which can be fatal. Mutations in the HBsAg coding sequence coinfected with HIV can accumulate HBsAg in hepatocytes and damage the host’s endoplasmic reticulum and DNA damage (Pollicino et al., 2014). With HIV-HBV coinfection, immune reconstitution inflammatory syndrome (IRIS) is developed more profoundly (Shahani and Hamill, 2016). Acute HBV infection in 90–95% of adults can develop a broad and multispecific cellular immune response that can eliminate the virus by producing protective antibodies against HBsAg (Mahoney, 1999).

Moreover, chronic HBV-infected patients can develop anti-HBe associated with aging and aminotransferase (ALT) elevation (Liaw et al., 1988; Fattovich et al., 1991). In a 10-year study of 1536 Alaskan natives with chronic hepatitis B, 70 and 7% cleared HBeAg and HBsAg, respectively (McMahon et al., 2001). However, they might remain inactive carriers with HBsAg particles with either low or undetectable levels of HBV DNA (Fattovich et al., 1986; de Jongh et al., 1992). These inert carriers can significantly reduce the mortality rate twofold, although increased ALT can lead to fibrosis progression (Fattovich et al., 1990). However, when coinfection occurs with HIV, the mortality rate increases significantly in HBV patients elevating chronic liver diseases. Among 1000 people a year, 14.2% of them suffer from several chronic liver diseases when coinfected compared with 0.8% of people with HBV infection only (Thio et al., 2002). One study suggested that HIV-infected people had a greater clearance rate of HBsAg and HBeAg due to the degree of immunosuppression (Bodsworth et al., 1991). However, other studies showed that HIV infection reactivates HBV and accelerates the loss of anti-HBs, increases levels of HBV DNA, and significantly lowers the ALT levels (Biggar et al., 1987; Gilson et al., 1997). Although lower ALT levels indicate less hepatocyte destruction, anti-HBV therapy’s effectiveness decreases in coinfected individuals. HIV coinfection has also been reported to create fibrosing cholestatic hepatitis, which leads to liver graft rejection in liver transplant recipients. An extremely high level of HBV antigen expression is observed in this specific liver disease due to a direct cytopathic effect (Davies et al., 1991; Xiao et al., 2008).

Although most studies show significant evidence of HBV infection acceleration due to HIV, the impact of HBV infection in HIV patients is still quite limited. A study found that among 80 gay men, 32 anti-HBc positive men progressed to get acquired immunodeficiency syndrome (AIDS) more rapidly (Eskild et al., 1992). Another study showed that HIV-HBV coinfected patients with AIDS had decreased survival than AIDS patients (Ockenga et al., 1997). The outcome of HBV infection varies according to the age and the current immune status of the host. For example, HBV infection persists in 50–90% of persons infected at birth or early childhood.

In contrast, fewer than 5% of HBV infections become chronic among HIV-uninfected adults (Yim and Lok, 2006). A multicenter-AIDS-cohort study suggested that coinfected patients had a mortality rate eight times higher than those singly infected. Moreover, the mortality related to hepatopathy due to hepatitis B has increased significantly since the highly active antiretroviral therapy (HAART), a treatment for HIV patients (Miailhes et al., 2007). Previous studies show that coinfection causes liver-related mortality 19 times that of a single infection (Thio et al., 2002). The mortality rate increased in individuals with lower CD4+ T-cell counts for the infection (Jaroszewicz et al., 2012; Figure 4).

### Human Immunodeficiency Virus-Hepatitis C Virus

Human immunodeficiency virus -Hepatitis C virus coinfected patients have higher HCV RNA loads and experience more rapid progression of HCV-related liver diseases than those without HIV infection (Hoofnagle and Di Bisceglie, 1991). Several studies suggested plenty of symptoms of the coinfection (Figure 4); however, these can vary over different factors, such as consumption of alcohol, age at acquisition of infection, race, sexual status, concomitant viral infection with HIV, time of illness, body mass index, and various genetic factors. HIV-HCV coinfected patients suffer from higher liver-related morbidity and mortality, non-hepatic organ dysfunction, and overall mortality than HCV single infected patients (Lo Re<suffix>III</suffix>, Kallan et al., 2014). Moreover, coinfection causes an increased rate of severe liver fibrosis and cirrhosis (Fierer et al., 2013; Kirk et al., 2013). A meta-analysis of eight separate studies found that HIV-HCV coinfected patients had approximately two times higher risk of cirrhosis diagnosed on liver biopsy and around six times higher risk of decompensated liver disease than HCV-infected patients (Thein et al., 2008; Tuyama et al., 2010). Coinfection can cause immune dysfunction, cytokine production, and cell apoptosis, leading to severe immunodeficiency (Körner et al., 2009; Kovacs et al., 2010). It causes HIV-related mitochondrial translocation-induced immune activation and causes severe liver damage (Kim and Chung, 2009; Rotman and Liang, 2009). HIV- and HCV-associated chronic inflammation leads to endothelial dysfunction (Bedimo et al., 2010). Moreover, coinfection also causes thrombocytopenia and osteoporosis (Marks et al., 2009). It is estimated that 90% of HIV patients with acute HCV will gradually develop chronic HBV, which shows a higher prevalence in coinfected patients than singly-infected (Collin et al., 2009). Other reports suggest that coinfection also increases the rate of related diseases such as cryoglobulinemia, hemophilia, diabetes mellitus, and kidney-related infections (Sulkowski, 2001). It is confirmed that HIV infection substantially impacts mortality among HCV-infected individuals, mainly due to HIV-induced immunodeficiency (Singal and Anand, 2009).

### Hepatitis B Virus-Hepatitis C Virus

Hepatitis B virus-Hepatitis C virus coinfection is more frequently found in several high-risk populations, for example, persons who inject drugs, patients on hemodialysis and undergoing organ transplantation, HIV-positive, and β-thalassemia (Pallás et al., 1999; Aroldi et al., 2005; Reddy et al., 2005). Tyson et al. stated that the independent associations with HBV coinfection compared with HCV single infection were age ≤50 years, male sex, positive HIV status, history of hemophilia, sickle cell anemia, or thalassemia, history of blood transfusion, cocaine, and other drug uses. At the same time, there was decreased risk in patients of Hispanic ethnicity (Tyson et al., 2013). With hidden hepatitis B, liver cirrhosis occurs in 33% of HCV coinfected patients and 19% of HCV carriers with undetectable HBV DNA (Fan et al., 2003). Superinfections cause the development of chronic hepatitis, for which the mortality rate is as high as 10% (Sterling and Sulkowski, 2004). Moreover, coinfection increases the rate of liver cirrhosis, chronic and fulminant hepatitis, and hepatocellular carcinoma (Figure 4).

## Diagnosis of Coinfection

### Human Immunodeficiency Virus-Hepatitis B Virus

All patients infected with HIV must be tested for HBV and HCV and vice versa, as the transmission route is the same. The HIV-negative patients, only infected with any of the hepatitis viruses, are required to test for alanine aminotransferase (ALT), aspartate aminotransferase (AST), hemoglobin, white blood cell count, platelets, HBeAg, HBe antibody (anti-HBe), HBsAg antibody (anti-HBs), CD4 count, and HBV DNA quantification in the serum (Audsley et al., 2020). HBV DNA levels are a significant marker to detect coinfection (Lok and McMahon, 2009). Spontaneous sero-reversion can occur due to having a low CD4 count (<200/mm3) and is also a prime marker for HIV-HBV coinfection. Therefore, HBV serological tests should be repeated among HIV-infected patients with prior positivity for HBsAb for the reemergence of HBV infection (European Association for the Study of the Liver, 2012). Anti-HBc can also be found in HIV-positive patients due to having past HBV infection (Gandhi et al., 2005). The occurrence of a past infection is often termed “Occult HBV infection,” which results in the presence of HBV DNA in the absence of HBsAg and has been reported in 2 to 10% of HIV-infected patients (Shire et al., 2007; Tsui et al., 2007). For HCC screening, coinfected patients with HBV-HIV should undergo serial liver ultrasound examinations and alpha-fetoprotein (AFP) serology every 6 months (European Association for the Study of the Liver, 2012). Severe conditions with HIV-HBV coinfection may require a liver biopsy. However, non-invasive measures, such as serum fibrosis markers and transient elastography, can help determine the degree of underlying fibrosis instead of biopsy (Moreno et al., 2009; Smith and Sterling, 2009). The markers suggest different parameters for detecting HIV-HBV coinfection depending on HBV infection types (Table 2).

**TABLE 2:** Serological markers of HIV-HBV, HIV-HCV, and HBV-HCV coinfection.

	HIV-HBV coinfection markers	HIV-HCV coinfection markers	HBV-HCV coinfection markers
Genomic level	↑ HBV DNA	↑ HCV RNA	↑ HBV DNA
T-cell count	↓ CD4 count (<200/mm3)	↓ CD4 and CD8 ratio	-
Antigen/Antibody	↑ HBsAb ↑ Anti-HBc	↑Anti-HCV	Delayed HBsAg ↓ HBs antigenemia
Related infection	Occult infection: ↑HBV DNA ↓HBsAg	-	HCV superinfection and chronic HBV infection: ↑ HBeAg ↓ HBsAg
Enzyme markers	↑ alpha-fetoprotein	↑ AST:APRI ↑ APRI score (>1.5) ↓ AST *p*-value (<0.0001)	↑ biphasic alanine aminotransferase

### Human Immunodeficiency Virus-Hepatitis C Virus

Reverse transcriptase-nested polymerase chain reaction assays (RT-PCR) must be conducted for the screening of the persistent HCV 5′ untranslated region (5′ UTR) for the diagnosis of HCV RNA in HIV patients (Panigrahi et al., 1994). Liver fibrosis progression is more rapid in the context of HIV-HCV coinfection and is associated with lower CD4 cell counts (Martin et al., 1989; Puoti et al., 2001; Martinez-Sierra et al., 2003). Non-invasive measures of liver fibrosis can be a reliable diagnosis for HIV-HCV coinfection. The alanine aspartyl transferase (AST)-to-platelet ratio index (APRI) has been validated as a good predictor of significant liver fibrosis in both HCV single and coinfection when compared with liver biopsy (Wai et al., 2003; Lackner et al., 2005). One study suggested that, in coinfection, APRI score > 1.5 was 100% specific and 52% sensitive for predicting significant fibrosis (Al-Mohri et al., 2005). Forrester et al. reported that AST and alanine aminotransferase (ALT) levels were significantly higher (*P* < 0.0001), and platelet counts were lower (*P* < 0.01) in HIV-HCV coinfected individuals than in patients infected with only HIV (Forrester et al., 2012). Other markers such as alpha-2-macroglobulin, apolipoprotein A1, haptoglobin, γ-glutamyl transpeptidase (GGT), and bilirubin are also screened primarily focuses on HCV patients (Table 2; Imbert-Bismut et al., 2001; Morali et al., 2007).

### Hepatitis B Virus-Hepatitis C Virus

Laboratory evaluation for coinfection with all possible viral causes, including HBV and HCV, is performed in patients presenting with acute hepatitis resulting from any of the two viruses. In case of superinfection of HCV or HBV, silent or occult HBV, all the necessary parameters and confirmatory tests such as HBV DNA testing by polymerase chain reaction (PCR) should also be performed when clinically indicated. In acute infection with HBV and HCV, patients showed delayed HBsAg appearance and a shorter HBs antigenemia than those with acute HBV alone (Mimms et al., 1993). According to an Italian investigation that showed active HBV-HCV indicators in 30 patients with HCV infection, the chronicity rates were equivalent to individuals with a single infection with either of the viruses. Biphasic alanine aminotransferase elevation was also observed in the case of HBV-HCV coinfection (Yan and Lee, 2005). Several reports have documented that *de novo* HCV superinfection in the setting of chronic HBV infection can result in HBeAg seroconversion and clearance of HBsAg.

Moreover, fulminant hepatic failure was significantly higher among patients with underlying HBV infection than those singly infected (23 vs. 3%) (Wu et al., 1994). Hence, every marker should be adequately detected with a specific protocol. On the other hand, occult hepatitis B (OHB) is defined as the presence of HBV DNA in serum and the liver tissue without detectable HBsAg with or without anti-HBc or anti-HBs outside the pre-seroconversion window period (Table 2; Bréchot et al., 2001).

## Prospective Treatments

### Human Immunodeficiency Virus-Hepatitis B Virus Coinfection

The most common treatment for HIV-HBV coinfection is HBV-active ART, usually tenofovir together with either lamivudine or emtricitabine. This treatment significantly decreases the rate of HBeAg and HBsAg antigens in infected individuals (Boyd et al., 2016). Drugs such as interferon alfa, peginterferon alfa, adefovir, lamivudine, entecavir, tenofovir, emtricitabine are used to prevent the coinfection (Table 3). As HIV infection can accelerate the progression of HBV-related liver disease, treatment of chronic hepatitis B is generally recommended for most HIV-infected patients with active HBV infection.

**TABLE 3:** Potential medications and their activity against HIV-HBV coinfected individuals.

Drug	Direct activity against HIV and HBV	For chronic HBV in HIV-infected patients	References
Interferon Alfa	No	No	Johnson et al., 2007
Peginterferon Alfa	Yes	No	Di Martino et al., 2002
Lamivudine	Yes	No	Benhamou et al., 1999; Dore et al., 1999
Emtricitabine	Yes	No	Lim et al., 2006; Saag, 2006
Adefovir	No	No	Hadziyannis et al., 2003; Thio, 2006
Tenofovir	Yes	Yes	Lada et al., 2004
Entecavir	Yes	Yes	Tenney et al., 2004; Pessôa et al., 2008

### Human Immunodeficiency Virus-Hepatitis C Virus Coinfection

Generally, HIV-HCV coinfection treatment focuses on eliminating a single infection first by decreasing the viral load alongside treating the secondary issues. A guideline is followed to treat HCV, but there is still a lack of information about HIV and HCV disease stage and viral load, HCV genotype, degree of hepatic fibrosis, and patient’s readiness to tolerate and adhere to treatment (Soriano et al., 2007). The treatments are more likely to target the current standard pegylated interferon plus ribavirin (pegIFN + RBV) medication, predictors of the treatment response, adverse events of anti-HCV therapy, and HAART as both HIV-HCV therapeutic agents (Table 4). The most effective treatment to date for HCV infection is direct-acting antiviral (DAA) therapy. First-generation DAAs include the NS3/4A serine protease inhibitor telaprevir and the NS5B RNA polymerase inhibitor sofosbuvir (Messina et al., 2015). Clinical trials demonstrated that using these DAAs in combination with peginterferon and ribavirin gave sustained virologic response (SVR) to HCV genotype 1 infection, up to 75% for telaprevir and 90% for sofosbuvir (Walker et al., 2015). The second-generation DAA regimens paritaprevir/ritonavir/ombitasvir, dasabuvir (3D), and sofosbuvir/ledipasvir were approved by the United States Food and Drug Administration (FDA) for patients with HCV genotype 1, as these all-oral regimens showed improved efficacy, safety, and tolerability when compared to first-generation protease inhibitor regimens (Pawlotsky, 2014). However, many long-term studies have shown that patients with compensated cirrhosis who achieved SVR with interferon (IFN)-based therapy showed efficacy at first, but various side-effects and limited virological effectiveness was seen with long-term treatment. However, with DAAs, patients with compensated cirrhosis achieve SVR rates over 95% (Afdhal et al., 2014; Poordad et al., 2014; Krassenburg et al., 2021). Moreover, high rates of cure have also been observed in groups including patients with cirrhosis and HIV coinfection (Hawkins et al., 2016). Many open-labeled studies demonstrated that DAAs such as ledipasvir and sofosbuvir for 12 weeks provided high rates of SVR in the patients coinfected with HIV-1 and HCV genotype 1 or 4 (Naggie et al., 2015). Another study suggested that treatment with the all-oral, interferon-free 3D-plus-ribavirin regimen resulted in high SVR rates among patients co-infected with HCV genotype one and HIV-1 (Sulkowski et al., 2015).

**TABLE 4:** Effects and side-effects of potential medications for HIV-HCV coinfected individuals.

Drugs	Therapeutic effects	Side effects	References
HAART	Decreases HIV replication in liver Decreases proinflammatory cytokines and necro-inflammatory activity	Increases Liver enzymes (LEE) and hepatotoxicity	Pascual-Pareja et al., 2009; Price et al., 2009
Protease inhibitors	Decreases HCV replication		Toma et al., 2009
Efavirenz	Decreases hepatotoxicity		Labarga et al., 2007
NNRTI + pegIFN + RBV	Decreases synergistic HCV replication *in vitro*	Increases Mitochondrial toxicity	Labarga et al., 2007; Toma et al., 2009
Simeprevir (SMV) + PR	Decreases hepatotoxicity		Jacobson et al., 2014
DAAs (Telaprevir/Sofosbuvir/Paritaprevir /Ritonavir/Ombitasvir)	Increase sustained virologic response upto 90%		Naggie et al., 2015; Wyles et al., 2015

### Hepatitis B Virus-Hepatitis C Virus Coinfection

Hepatitis B virus-Hepatitis C virus coinfection is more complex than a single infection with HBV or HCV alone. Hence, the general approach for treatment is first to identify the dominant virus, treat that virus as a single infection, and then monitor for reactivation of the other virus. Coinfection is typically treated with a nucleotide analog (such as lamivudine, entecavir, or tenofovir) along with PegIFN (Sulaiman, 1989). It is 35% effective in HBV and 50–60% effective in HCV when combined with ribavirin (Crockett and Keeffe, 2005). Moreover, direct-acting antiviral (DAA) is also used, which gives 90% efficacy for inhibiting HCV but no response for HBV (Calvaruso et al., 2018). It is recommended to vaccinate HBV patients because studies showed 60% efficacy to vaccinated cirrhotic patients (Roni et al., 2013). Liu et al. (2014) assessed that peg-IFN/RBV therapy is not only safe and effective but translates into significant clinical benefits such as reduction in liver-related complications and improved patient survival. However, in terms of viral dominance, it is not common to have codominance of both viruses. Hence, there is either HBV dominance, which means high HBV DNA levels and low HCV RNA levels, or HCV dominance, defined by the high HCV RNA levels and absent HBV DNA (Konstantinou and Deutsch, 2015). HBV dominance is likely to be more common in Asian patients, while HCV dominance is seen among North American coinfected and European patients (Nguyen et al., 2011). Therefore, it is suggested to diagnose the disease properly and determine the dominant side before treating a coinfected patient. An Italian study reported that 9 million IU of standard IFN 3 times weekly for 3 months could clear HCV in 31% of HCV-HBV coinfections (Villa et al., 2001). As previously mentioned, peg-IFN is one of the first-line choices for treating chronic HBV infection, and does not cause any side effects toward coinfected patients (Bihl et al., 2010). However, till present, no data have been published regarding the efficacy of direct-acting antivirals (DAAs) in combination with peg-IFN plus RBV or with IFN-free regimens in treating patients with chronic HBV-HCV coinfection.

## Conclusion and Future Directions

Coinfection with HBV or HCV is common among HIV-infected individuals due to their similar modes of transmission. Any coinfection can reportedly lead to an increased risk of morbidity and mortality. Overall, the prevalence of hepatitis B and hepatitis C, with HIV coinfection, is 50.3%. Among these, HIV-HBV is 8.4%, HIV-HCV is 35.4%, and HBV-HCV is 5%. The characteristics of HIV-infected individuals can differ according to the coinfecting hepatitis viruses that can change their strains over time. Therefore, frequent screening and monitoring should be performed in both coinfected and singly infected patients due to the possibility of developing another secondary infection if measurable steps are not taken. In addition, one must get tested if their partner is positive for any infection or assume any symptoms.

Treatments for coinfected patients are complex due to the interaction of the two viruses and the potential for reactivation of one virus with antiviral therapy directed against only the other virus. Therefore, coinfected patients must undertake single infection treatment concurrently with dual antiviral therapy. Until today, highly active antiretroviral therapy (HAART) has been a breakthrough for treating patients with HIV, as the infection currently does not have any effective vaccination. HCV prevention is also based on direct-acting antiviral (DAA) treatments for short-term and curative measures.

Vaccination is given to prevent HBV with three consecutive doses for the long term. Early remedies for acute infection are also highly encouraged. However, several preventive strategies must be followed to prevent and control these blood and fluid-borne viral diseases. It is highly advisable to avoid percutaneous exposure incidents, such as needle injuries, sharp injuries, and splashes leading to exposure of the skin or mucosa to blood. Drug abuse and sharing the same needle or syringe are also high-risk behaviors toward these diseases. Safe sex and special care for pregnant women are recommended to prevent disease occurrence. Besides conventional therapy and treatments, CRISPR-Cas9-based antiviral strategies can also be implied to avoid hepatitis coinfection due to its relative versatility, specificity, and ease of use. Vaccines against HCV and HIV should be further studied and implemented at the earliest convenience. Polyvalent vaccines for these viral coinfections can also be an escalating preventive measure for the prospective times. Although current diagnostic technologies can detect viruses, they still rely on practical labor, expensive and limited resources. However, if nanotechnology (metal/inorganic nanoparticles, carbon nanotubes) combined with microfabrication can be used in diagnostic and therapeutic settings, the healthcare systems will undergo a revolutionary shift. These technologies with miniatured sensors can be used to easily detect viruses from an ultra-low volume of blood, serum, and plasma, allowing highly accurate diagnosis. However, further extensive studies should be conducted to diagnose and treat the coinfection of these blood-borne viral diseases.

## Author Contributions

MGH designed and supervised the manuscript. SaS, YA, RA, PK, GS, TR, RS, and ShS searched and collected the literature. SaS and YA wrote the preliminary draft manuscript. YA and TR designed the graphs. MSI and MGH reviewed the preliminary draft manuscript. CZ and MGH edited, revised, and finalized the manuscript. All authors read and approved the manuscript.

## Conflict of Interest

The authors declare that the research was conducted in the absence of any commercial or financial relationships that could be construed as a potential conflict of interest.

## Publisher’s Note

All claims expressed in this article are solely those of the authors and do not necessarily represent those of their affiliated organizations, or those of the publisher, the editors and the reviewers. Any product that may be evaluated in this article, or claim that may be made by its manufacturer, is not guaranteed or endorsed by the publisher.
