# Corrigendum: Insights into the coinfections of human immunodeficiency virus-hepatitis B virus, human immunodeficiency virus-hepatitis C virus, and hepatitis B virus-hepatitis C virus: prevalence, risk factors, pathogenesis, diagnosis, and treatment

**DOI:** 10.3389/fmicb.2023.1253124

**Published:** 2023-08-01

**Authors:** Sagarika Shahriar, Yusha Araf, Rasel Ahmad, Pravakar Kattel, Ganga Sagar Sah, Tanjim Ishraq Rahaman, Rahila Zannat Sadiea, Shahnaj Sultana, Md. Sayeedul Islam, Chunfu Zheng, Md. Golzar Hossain

**Affiliations:** ^1^Biotechnology Program, Department of Mathematics and Natural Sciences, BRAC University, Dhaka, Bangladesh; ^2^Department of Genetic Engineering and Biotechnology, School of Life Sciences, Shahjalal University of Science and Technology, Sylhet, Bangladesh; ^3^Department of Microbiology and Hygiene, Bangladesh Agricultural University, Mymensingh, Bangladesh; ^4^Department of Biotechnology and Genetic Engineering, Faculty of Life Sciences, Bangabandhu Sheikh Mujibur Rahman Science and Technology University, Gopalganj, Bangladesh; ^5^Department of Biological Sciences, Graduate School of Science, Osaka University, Osaka, Japan; ^6^Department of Microbiology, Immunology and Infectious Diseases, University of Calgary, Calgary, AB, Canada

**Keywords:** coinfection, HIV, HBV, HCV, prevalence, risk factors, pathogenesis

In the published article, there was an error in affiliation 1. The following affiliation should be removed:

“Department of Immunology, School of Basic Medical Sciences, Fujian Medical University, Fuzhou, China.”

The authors apologize for this error and state that this does not change the scientific conclusions of the article in any way. The original article has been updated.

